# Continuous-Flow Chemistry and Photochemistry for Manufacturing of Active Pharmaceutical Ingredients

**DOI:** 10.3390/molecules27238536

**Published:** 2022-12-04

**Authors:** Pavlína Horáková, Kamila Kočí

**Affiliations:** 1 Institute of Environmental Technology, CEET, VŠB-Technical University of Ostrava, 708 00 Ostrava, Czech Republic; 2 TEVA Czech Industries s.r.o., 747 70 Opava, Czech Republic

**Keywords:** active pharmaceutical ingredients, flow chemistry, photochemistry

## Abstract

An active pharmaceutical ingredient (API) is any substance in a pharmaceutical product that is biologically active. That means the specific molecular entity is capable of achieving a defined biological effect on the target. These ingredients need to meet very strict limits; chemical and optical purity are considered to be the most important ones. A continuous-flow synthetic methodology which utilizes a continuously flowing stream of reactive fluids can be easily combined with photochemistry, which works with the chemical effects of light. These methods can be useful tools to meet these strict limits. Both of these methods are unique and powerful tools for the preparation of natural products or active pharmaceutical ingredients and their precursors with high structural complexity under mild conditions. This review shows some main directions in the field of active pharmaceutical ingredients’ preparation using continuous-flow chemistry and photochemistry with numerous examples of industry and laboratory-scale applications.

## 1. Introduction

In recent years, there has been an explosion in the use of flow chemistry and photochemistry in organic synthesis. Flow chemistry allows the continuous synthesis of the target compound under controlled and mild conditions [1,2]. Synthesis under continuous-flow conditions has become an enabling technology for improving synthetic efficiency through automation and process optimization [3]. It enables easy coupling of individual reaction steps without the need for isolation and purification of intermediates [4,5]. A multistep synthetic sequence in the flow mode relies on several reactors, which is in line with the great benefit that intermediates are not isolated but directly transferred to the next flow reactor [6]. There are several advantages over batch methods—for example, better mixing, efficiency, heat transfer and safety [1]. Conversion and selectivity are critically dependent on the quality of mixing when the rate of reaction is higher than the speed of mixing. Mechanisms of mixing can be molecular in scale, or external forces can be applied [7]. Due to the high surface area-to-volume ratios, heat can be applied and removed efficiently, allowing precise control of the reaction temperature. The stability of small-diameter capillary reactors under high pressures allows the reactors to operate safely under high pressures and temperatures. This can be very useful for reducing safety hazards when executing exothermic reactions or even reactions which proceed via highly unstable or explosive intermediates [8]. Flow reactors are devices where chemical reactions are performed in narrow channels [2]. They mostly consist of chemically resistant capillaries or microchannels and of devices such as pumps or mixers [9]. Flow devices can be easily combined with other technologies, leading to improved efficiency. Typical enabling technologies joined to flow chemistry are microwave irradiation, 3D printing, electrochemistry, inductive heating and light activation–photochemistry [10,11].

Using light to accelerate a chemical reaction is one of the most promising approaches to accessing new chemical transformations in a more effective and sustainable way [4]. The photon is considered as a green reagent that is absorbed without leaving any residue. Light-induced activation offers a way to prepare highly reactive entities under environmentally friendly conditions. These activations can be often supported by utilizing a photocatalyst or photosensitisors [12]. Photocatalysts can be homogeneous organic dyes or transition metal complexes, which are often very costly. Heterogeneous semiconductors in various forms can be applied [13]. On the other hand, the appropriate equipment (lamps with emission spectra which overlay the absorption spectra of the irradiated compound, power supply, heat transfer device and others) are required for a successful photoreaction [14]. However, developing photochemistry, especially at the industrial scale where significant productivity is required, continues to be a challenge [15,16].

While the perceived benefits of using flow chemistry vary across the different stages of the pharmaceutical industry [17], it has become a high-impact tool when applied appropriately. Flow chemistry has become a popular tool in the pharmaceutical industry [18]. The majority of companies have invested into this technology, in many cases having entire manufacturing plants dedicated to this technology [5]. A more recent trend, however, is to implement flow chemistry in a drug discovery setting [19,20].

In order to evaluate the current standing of this field, we review several flow and photochemistry syntheses of active pharmaceutical ingredients (APIs) conducted by scientists from both academia and industry and compare them to the batch preparations. This should give the reader an overview of advantages of synthesis under either photo or flow conditions compared to batch preparation regardingcapacity, reaction time, safety, heat transfer and others.

## 2. Continuous-Flow Chemistry

### 2.1. Ibuprofen

Ibuprofen (isobutylphenyl propionic acid), **6**, is a nonsteroidal anti-inflammatory drug that is widely used to alleviate pain, fever and inflammation. Among several benefits ensured by continuous-flow operations, there is the possibility of speeding up the chemical process to achieve high throughput.

**Scheme 1:**
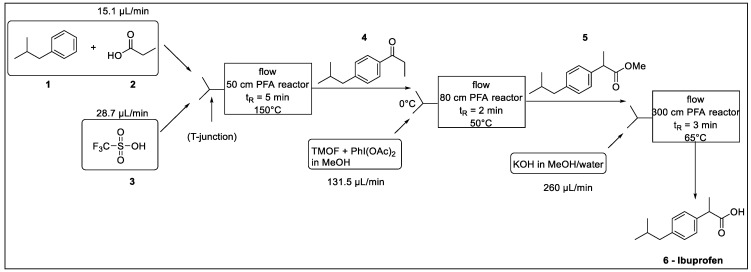
Ibuprofen flow synthesis: **1**—isobutylbenzene, **2**—propionic acid, **3**—triflic acid, **4**—1-(4-isobutylphenyl)propan-1-one, **3**—methyl 2-(4-isobutylphenyl)propanoate, **6**—ibuprofen.

In 2009, a flow synthesis of this high-volume drug was reported by the McQuade’s group [21]: a three-step approach to synthesizing ibuprofen (Scheme 1) using microreactor technology. A fully continuous process was intended, in which only the final sequence of purification was to be performed off-line. Each of the individual steps was first optimized in flow with the following steps in mind. The initial step was Friedel–Craft acylation of isobutylbenzene **1** (flow rate: 15.1 µL/min) with propionic acid **2** (flow rate: 15.1 μL/min) in the presence of excess triflic acid **3** (flow rate: 28.7 µL/min). The transformation was found to work very well, and the acid catalyst was not an issue in the subsequent 1,2-aryl migration step. This was catalyzed by an iodine reagent PhI(OAc)_2_ and conducted in trimethyl orthoformate (TMOF) and methanol (mixture flow rate: 131.5 µL/min). The direct hydrolysis of the resulting methyl ester **5** with an excess of base completed the flow synthesis of ibuprofen. Work-up proceeded via acidification and repeated washes with ether and water, then by treatment with activated charcoal; and lastly, recrystallization was performed manually to yield pure (99%) ibuprofen, **6**. Overall, this pioneering work allowed for the synthesis of ibuprofen in only 10 min residence time, with a yield of 51%; thus, the productivity was 9 mg/min [15].

**Scheme 2:**
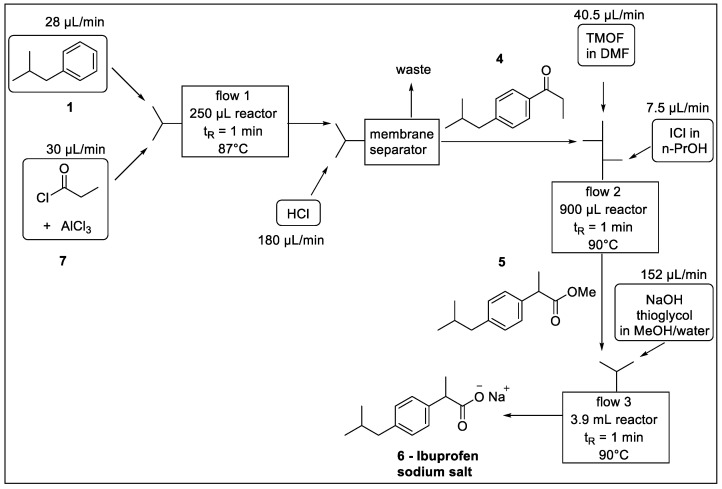
Scheme of the three-minute ibuprofen synthesis from Jamison (2015) [16]: **1**—isobutylbenzene, **4**—1-(4-isobutylphenyl)propan-1-one, **5**—methyl 2-(4-isobutylphenyl)propanoate, **6**–ibuprofen (sodium salt), **7**—propionyl chloride.

Extending a previously reported procedure, Jamison and Snead [16] discussed a continuous-flow synthesis. In 3 min only, API ibuprofen, **6,** was prepared starting from very simple building blocks and inexpensive reagents with an overall yield of 83% (three bond-forming steps, one work-up, and an online extraction) (Scheme 2). Reactors were made from coils of perfluoroalkoxylkane (PFA) tubing (inner diameter: 0.03 inches) immersed in a heated oil bath. Each reagent entered at a different flow rate, and even though highly reactive chemicals under harsh reaction conditions were utilized, the flow methodology guaranteed a high level of safety. Ibuprofen (**6**) could be produced under flow conditions for several hours with an output of 8.1 g/h [22].

### 2.2. Warfarin

Warfarin is an effective antithrombotic coumarin agent. It affects four blood coagulation proteins that act sequentially to produce thrombin. Coumarin therapy decreases the bioactivity of these proteins and so decreases the rate at which blood clots [23].

**Scheme 3:**
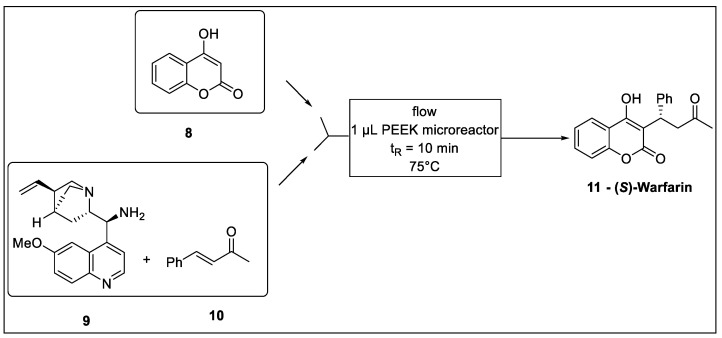
Enantioselective continuous-flow synthesis of (*S*)-warfarin: **8**—4-hydroxy-coumarin, **9**—cinchona-derived primary amine catalyst, **10**—benzalacetone, **11**—warfarin.

The continuous synthesis of (*S*)-warfarin was demonstrated by Porta et al. (2015) [11] using the nucleophilic addition of 4-hydroxy-coumarin **8** to benzalacetone **10** catalyzed by cinchona-derived amine **9** (10 mol%) with trifluoroacetic acid as a cocatalyst in dioxane (Scheme 3). This was the most straightforward methodology suitable for flow microreactors. In order to optimize the conditions, a polyetheretherketone (PEEK) microreactor (inner diameter: 0.58 mm; l = 189 cm) coiled in a bundle and immersed in a preheated oil bath was used. Different combinations of flow rates and reaction temperatures were investigated, leading to optimum reaction conditions: 75 °C and 1 µL/min flow rate (10 min residence time). This led to the formation of (*S*)-warfarin (**11**) with 61% conversion and 93% of enantiomeric excess (*ee*)—determined in a crude mixture (the product was not isolated). In this case, the scaling problem was observed. When a 500 µL reactor was used, the best conditions found in the smaller microreactor were not reproducible—the yield was quite low. This was solved by a “numbering-up” technique by connecting four same-dimension microreactors in parallel (flow rate: 4 µL/min; residence time (*t_R_*) 10 min in each reactor) with utilization of a stainless-steel splitter. At the end, warfarin (**11**) was isolated in 36% yield and 91% *ee* (productivity not stated) [22]. This is proof that a small microreactor can be utilized to find the best reaction conditions very quickly.

### 2.3. Atropine

Atropine is a drug occurring in nature in the *Belladonna* plant. Atropine can be administered via injection, eye drops, or in oral form to relax muscles by inhibiting nerve responses [24].

The first total synthesis of atropine was reported by Landenburg in 1879 [25]. However, the current industrial manufacturing process of atropine still relies mainly on herbal extraction [26].

**Scheme 4:**
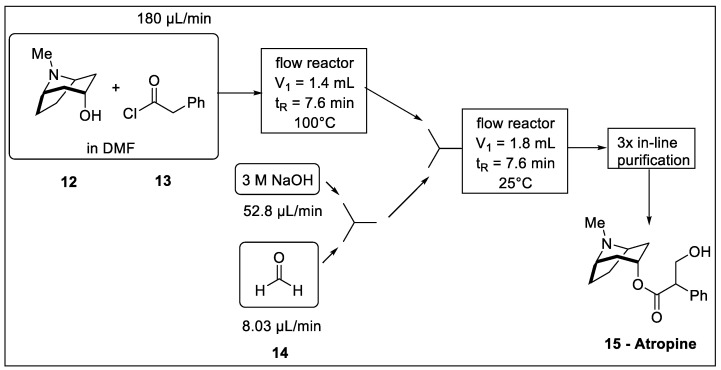
First flow formation of atropine: **12**—tropine, **13**—phenylacetyl chloride, **14**—formaldehyde, **15**—atropine.

The first flow-formation of atropine was originally published in 2015 [27] (Scheme 4), providing the target compound in >98% purity and only 8% overall yield in two steps (productivity 48 mg/h). The synthesis was developed using a commercially available flow system performed of PFA tubing (inner diameter: 0.03 inches). The first step was the reaction of tropine **12** with phenylacetyl chloride **13** in DMF at 100 °C with the flow rate of 180 μL/min and residence time *t_R_* = 7.6 min in a 1.4 mL reactor. The second step consisted of the reaction of the mixture of formaldehyde **14** with an aqueous solution of NaOH and the intermediate from the previous step at room temperature with residence time *t_R_* = 7.6 min in a loop of 1.8 mL volume at rate 52.8 μL/min. After the preparation, the sequential in-line liquid–liquid separations of impurities from the desired product followed.

**Scheme 5:**
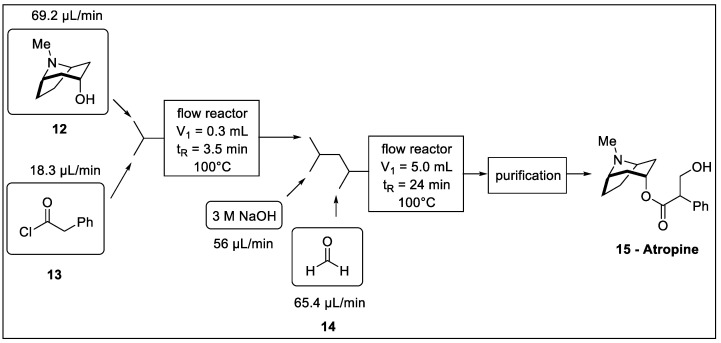
Atropine flow synthesis: **12**—tropine, **13**—phenylacetyl chloride, **14**—formaldehyde, **15**—atropine.

In 2017, the continuous-flow synthesis of atropine was demonstrated by Bédard et al. [28], which consisted of two flow reactions (Scheme 5). The first was the esterification of tropine **12** (in dimethylformamide) and neat phenylacetyl chloride **13** at 100 °C (residence 3.5 min and total flow rate: 87.5 µL/min) to form hydrochloride of the tropine ester, which is released in a free form by the addition of sodium hydroxide. In the second stage, the aldol addition of formaldehyde (**14**) to the tropine ester at 100 °C (*t_R_* = 24 min and total flow rate: 209 µL/min) forms the atropine (**15**). The two-step sequence to obtain atropine **15** was improved, and a yield of 22% was obtained (productivity: 996 mg/h) under optimum conditions [29]. The flow system consisted of PFA tubing with an inner diameter of 0.03 inches. The first flow system (2015) contained three different in-line liquid–liquid separations, and the revised system included only one separation [11].

### 2.4. Ketamine

Ketamine was originally used as an ingredient for anesthetic preparations, although strong dissociative side-effects led to its progressive withdrawal from the human pharmacopeia. Its unique antidepressant activity (even for multiresistant severe depressive disorders) was discovered later [30].

**Scheme 6:**
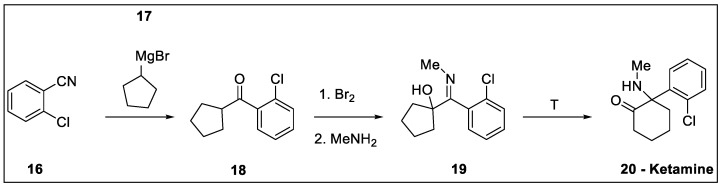
Parke–Davis and Company’s 1956 synthesis of ketamine: **16**—*o*-chlorobenzonitrile, **17**—cyclopentylmagnesium bromide, **18**—(2-chlorophenyl)(cyclopentyl)methanone, **19**—(2-chlorophenyl)(methylimino)methylcyclopentan-1-ol, **20**—ketamine.

The first batch synthesis (10 g scale) of ketamine (**20**) was reported by Parke-Davis and Company [31] in 1956 (Scheme 6). The synthesis started from *o*-chlorobenzonitrile **16,** which yielded ketone **18** after treatment with Grignard reagent **17**. Subsequent bromination, followed by imine formation and bromide hydrolysis, gave imine **19**. Heating of imine **19** resulted in the formation of ketamine (**20**). The source does not indicate the percentage yield [32].

**Scheme 7:**
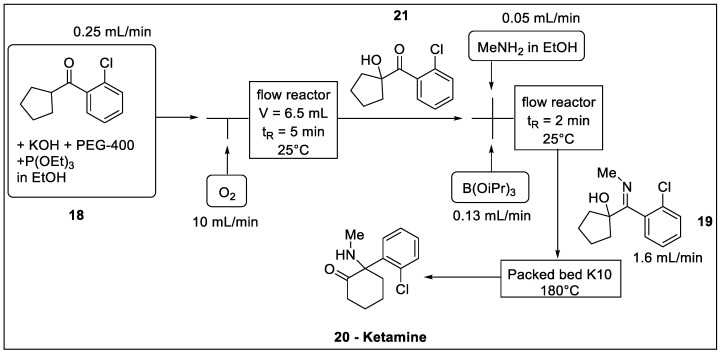
Flow synthesis of ketamine: **18**—(2-chlorophenyl)(cyclopentyl)methanone, **19**—(2-chlorophenyl)(methylimino)methylcyclopentan-1-ol, **20**—ketamine, **21**—2-(2-chlorophenyl)-2-(methylamino)cyclohexan-1-one.

Monbaliu et al. have recently reported [33] a novel continuous-flow procedure for the efficient and sustainable preparation of **20** (Scheme 7). The strategy features two steps under continuous-flow conditions, hydroxylation (step 1–residence time: 5 min, flow rate 0.25 mL/min) and imination (step 2–residence time (*t_R_*) 2 min, total flow rate 0.43 mL/min; 99% conversion; 97% selectivity). The final thermolysis relying on a packed-bed column of Montmorillonite K10 as a heterogeneous catalyst led to **20** with good quantitative conversion (70%; productivity not stated) and excellent selectivity (93%) [34]. Each step can be either run independently or be chained as a single reactor network.

### 2.5. Imatinib

Imatinib is a potent and selective inhibitor of the protein tyrosine kinase and is approved for the treatment of chronic myeloid leukemia and gastrointestinal stromal tumors [35].

**Scheme 8:**
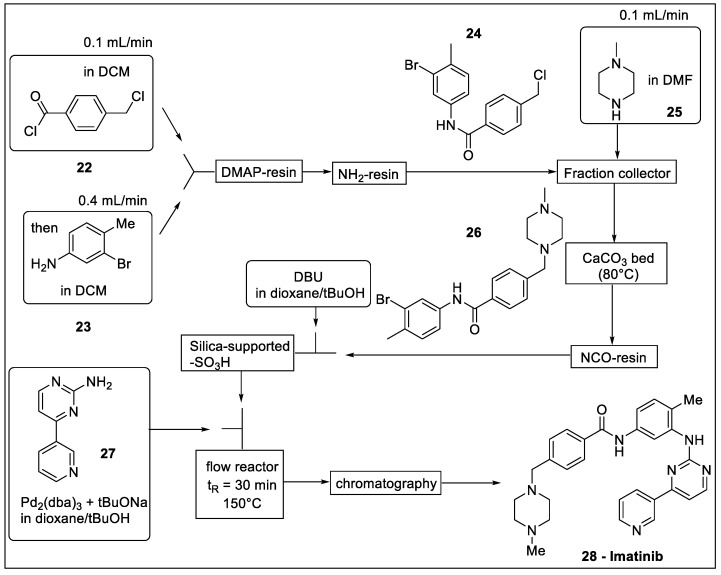
Flow synthesis of imatinib by Ley and co-workers: **22**—4-(chloromethyl)benzoyl chloride, **23**—3-bromo-4-methylaniline, **24**—*N*-(3-bromo-4-methylphenyl)-4-(chloromethyl)benzamide, **25**—1-methylpiperazine, **26**—substituted benzamide **27**—4-(pyridin-3-yl)pyrimidin-2-amine, **28**—imatinib.

Ley et al. reported [36] the synthesis of imatinib **28** and described a multistep flow process using in-line purification strategies (Scheme 8). All steps were conducted in a tubular flow loop or cartridges packed with either reagents or cartridges to obtain a clean product. The system contained a crucial in-line solvent switch that allows reaction solvents to be changed as a part of the continuous process to limit the number of human operations. The synthesis consisted of a reaction between benzoyl chloride **22** and methylaniline **23**, where their product **24,** after a reaction with methylpiperazine **25** and passage through various modified resins, was finally transferred to **28** after 30 min residence time in a flow reactor at 150 °C. After all, the product was obtained in 32% overall yield and 95% purity with the reagent flow 0.1 or 0.4 mL/min.

**Scheme 9:**
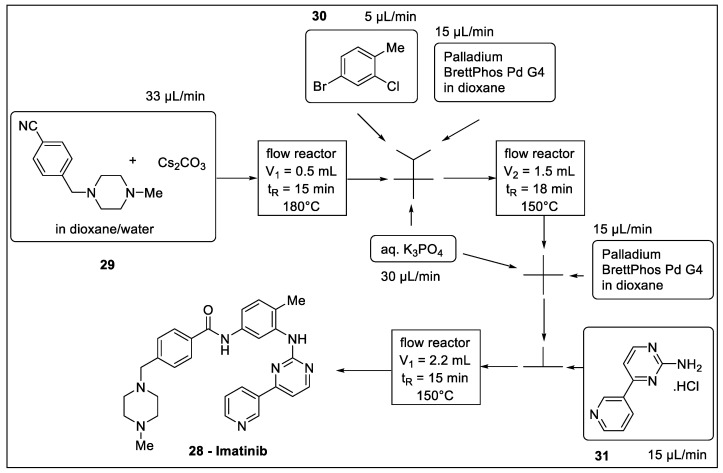
Flow synthesis of imatinib: **29**—(4-methylpiperazin-1-yl)methyl benzonitrile, **30**—4-bromo-2-chloro toluene, **28**—imatinib, **31**—2-aminopyrimidine hydrochloride.

Recently, Jamison et al. went a step further [37] by performing a one-flow synthesis of imatinib **28** without a solvent switch (Scheme 9). The API itself and several analogues were prepared in three chemical transformations, which were optimized separately in flow and combined in a final single setup. The synthesis of imatinib **28** started with the hydration of nitrile **29** under the flow with the residence time of 15 min at 180 °C. The next steps were palladium-catalyzed C-N cross-coupling at 150 °C. This one-flow approach led to a moderate yield of 58% (productivity 0.63 mmol/h) in the preparation of imatinib **28** with a residence time of 48 min in total via three-step synthetic sequence [38]. Each reagent had at a different flow rate, which varied from 5 to 143 µL/min. The flow reactors were stainless-steel coils with a 0.03 inch-inner diameter. The authors claim that to the best of their knowledge, the system delivers the highest production rate of imatinib **28**, and packed-bed apparatuses, in-line purifications, and solvent exchanges between individual steps were not required.

### 2.6. Rufinamide

Rufinamide is an antiepileptic drug, which is used with other medications to control seizures in people who have Lennox–Gastaut syndrome (a severe form of epilepsy). Rufinamide is in a class of medications called anticonvulsants. It works by decreasing abnormal excitement in the brain [39].

**Scheme 10:**
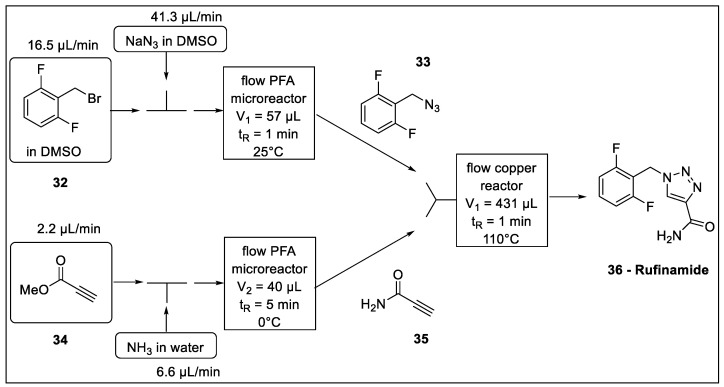
Flow synthesis of rufinamide: **32**—difluorobenzyl bromide, **33**—2-(azidomethyl)-1,3-difluorobenzene, **34**—methyl propiolate, **35**—propiolamide, **36**—rufinamide.

Rufinamide is conventionally prepared in batches [40,41]. Nevertheless, comparisons with flow approaches have been made [42,43]. Most of the synthetic routes relied on the initiation of the sequence from the benzyl azide intermediate **33**, which is a highly energetic substance. Jamison and coworkers [44] explored reducing the safety risks by preparing the organic azide **33** under the flow conditions (Scheme 10); thus, it was not necessary to isolate it. Each step was performed under a different flow rate of the reactants, which varied from 2 to 41 µL/min. The challenge was to combine both of the intermediates and to conduct the [3+2] cycloaddition in the next reactor. A solid formation was presumed, but it was not observed. On the other hand, only <10% conversion was obtained in PFA tubing. Inspired by Bogdan and Sach [45], the authors then used seamless copper tubing (inner diameter 0.03 inch) at an elevated temperature to observe 82% conversion to rufinamide (**36**). After lowering the temperature to 110 °C, the conversion was 98% and yield 92% (productivity 217 mg/h) at the residence time of 6 min in total for all the three steps.

**Scheme 11:**
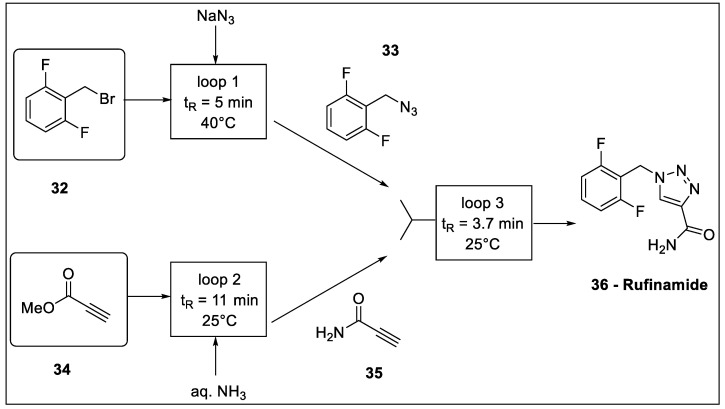
Convergent-flow synthesis of rufinamide utilizing radial system: **32**—difluorobenzyl bromide, **33**—2-(azidomethyl)-1,3-difluorobenzene, **34**—methyl propiolate, **35**—propiolamide, **36**—rufinamide.

**Scheme 12:**
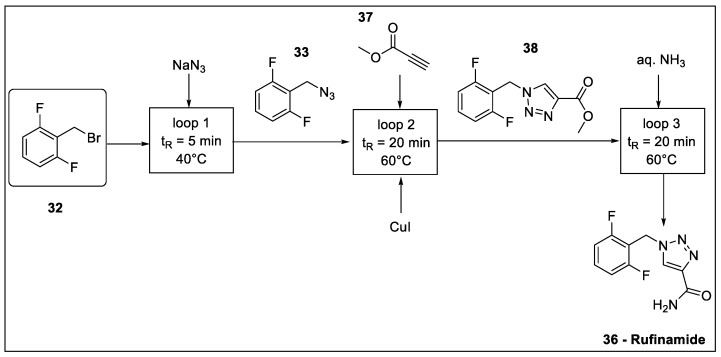
Linear Flow synthesis of rufinamide utilizing radial system: **32**—difluorobenzyl bromide, **33**—2-(azidomethyl)-1,3-difluorobenzene, **36**—rufinamide, **37**—methyl propiolate, **38**—methyltriazole carboxylate.

Gilmore and co-workers [46] demonstrated novel automated synthesis of rufinamide. A novel radial system, in which the reaction parameters can be changed by applying smart system loops via a digital interface, was described. The reactors were made of PFA, and the various components can be independently controlled. Utilizing this methodology, the authors synthesized rufinamide (**36**) in a convergent (Scheme 11) and linear sequence (Scheme 12). The convergent method provided **36** in 88% yield (239.4 mg/h) [38] via a three-step synthesis. Conditions for loop 1 were: residence time (*t_R_*) 5 min and total liquid flow rate: 3.6 mL/min; for loop 2: residence time 11 min and total liquid flow rate: 1.8 mL/min; and for loop 3: residence time 3 min 42 s and total liquid flow rate: 5.4 mL/min.

The linear method provided **36** in 83% yield (16.5 mg/45 min) via a three-step synthesis. Conditions for loop 1 were: residence time (*t_R_*) 5 min and total liquid flow rate: 3.6 mL/min; loops 2 and 3 ran under the same conditions of 20 min residence time and flow rate 0.8 mL/min in a linear set-up.

### 2.7. (−)-Oseltamivir

Oseltamivir is an oral anti-viral drug used for the treatment of acute, uncomplicated influenza types A and B by acting as a neuraminidase inhibitor [47].

A batch one-pot synthesis of (−)-oseltamivir was described in 2013 by Hayashi and co-workers [48]. They simply added the reagents sequentially without evaporation or a solvent swap. It takes 57 h to complete the whole synthesis, although it is a one-pot procedure. The product was obtained in 36% yield. 

**Scheme 13:**
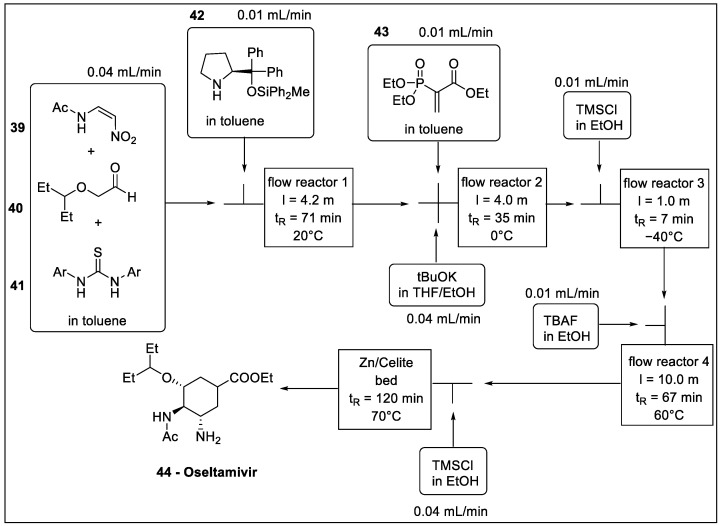
One-flow approach in (−)-oseltamivir synthesis: **39**—*N*-(2-nitrovinyl)acetamide, **40**—2-(pentan-3-yloxy)acetaldehyde, **41**—thiourea, **42**—diphenylmethyl pyrrolidine, **43**—ethyl 2-(diethoxyphosphoryl)acrylate, **44**—oseltamivir.

In their next work [49], after an extensive optimization, the authors introduced the preparation of (−)-oseltamivir (**44**) under the flow conditions (Scheme 13) consisting of five units. Four reactors were made of Teflon tubes; the last was a zinc/celite-packed bed. First, an asymmetric Michael reaction of aldehyde **40** with nitroalkene **39** was performed, followed by the second Michael reaction with phosphoryl acrylate, **43**. Then, an intramolecular reaction was performed, and lastly, the nitro group was reduced to obtain (−)-oseltamivir within 310 min for an overall yield of 13% (58 mg/15 h) at the flow rate 0.1 or 0.4 mL/min. Even though the Hayashi group successfully developed a continuous-flow synthesis of oseltamivir, the overall yield and the stability of the zinc/celite reactor (used in the last step) takes only approximately 5 h because zinc activity gradually decreased over a long period [38].

### 2.8. Linezolid

Linezolid is a drug used as the last line of defense against multi-drug-resistant Gram-positive bacteria [50].

**Scheme 14:**
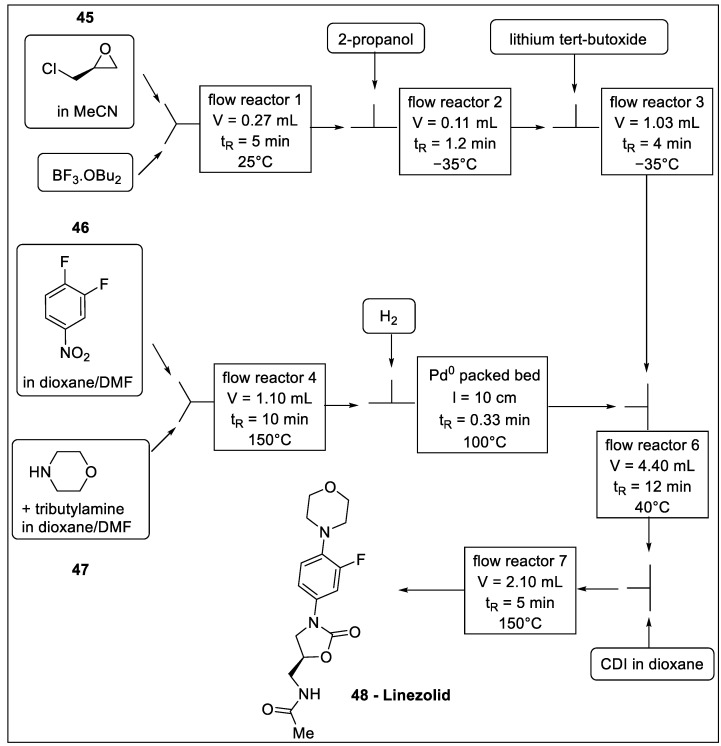
Fast-flow synthesis of Linezolid: **45**—(+)-epichlorhydrin, **46**—3,4-difluoronitrobenzene, **47**—morfoline, **48**—linezolid.

The existing batch synthesis of linezolid is rather time consuming (>60 h), but the material is obtained at a 72% yield [51]. Jamison and co-workers [52] developed a seven-step synthesis, which works in a fully continuous manner, through which a fast-flow synthesis of linezolid **48** has become available (Scheme 14). The synthesis in high purity PFA tubing reactors (inner diameter: 0.04 inch) starts from (+)-epichlorhydrin **45** and aniline building blocks, which are formed by reaction of 3,4-difluoronitrobenzene **46** and morpholine **47**. The epoxide intermediate is nucleophilically opened by reaction with carbonyldiimidazole (CDI); and finally, acidic hydrolysis provided **48** in an isolated overall yield of 73%, corresponding to a throughput of 816 mg/h. The total residence time for the seven-step sequence is 27 min (flow rate 55 µL/min or 186 µL/min respectively), which is significantly shorter than those of the reported batch procedures [38].

### 2.9. Lomustine

Lomustine is a widely used anticancer agent for the treatment of brain tumors. In the U.S., the patent for lomustine has expired, but only one company—CordenPharma—manufactures it [53]. This led to a >1500% price hike from 50 USD to 768 USD per capsule [54].

**Scheme 15:**
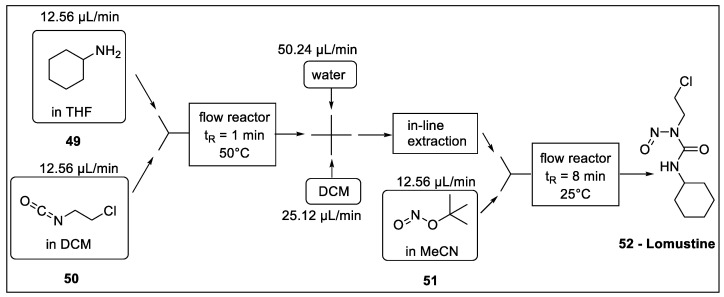
Lomustine flow synthesis: **49**—cyclohexylamine, **50**—1-chloro-2-isocyanatoethane, **51**—*tert*-butyl nitrite, **52**—lomustine.

Thompson and co-workers [55] investigated a rapid, continuous one-flow system (Scheme 15) to obtain **52** within two chemical steps and an in-line extraction. The synthesis was initiated by carbamoylation of cyclohexylamine, **49**, with 1-chloro-2-isocyanatoethane, **50**, followed by nitrosation with *tert*-butyl nitrite, **51**. With this one-flow system made of fluorinated ethylene propylene (FEP) tubing (0.8 mm diameter), **52** was obtained in an overall yield of 63% with 9 min residence time in total. Each step was performed under a different flow rate for the reactants, which varied from 12 to 50 µL/min [38]. Using this method, 110 mg/h of Lomustine can be produced.

### 2.10. Rolipram

Rolipram is an important γ-aminobutyric acid (GABA) derivative with anti-inflammatory properties [56]. Moreover, rolipram is known to be a possible antidepressant and has been reported to have immunosuppressive and antitumor effects [57].

**Scheme 16:**
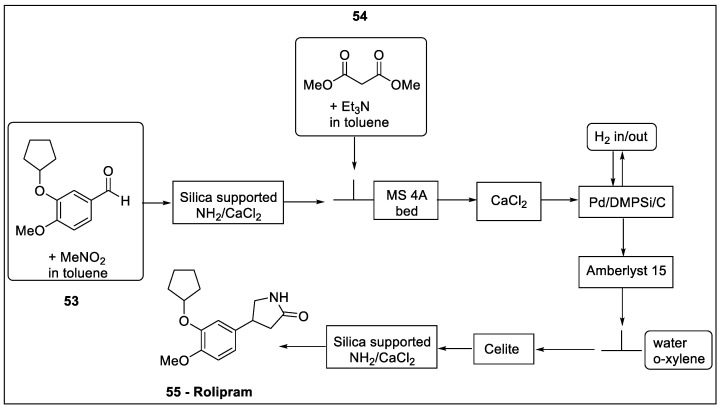
Continuous-flow synthesis of rolipram: **53**—3-(cyclopentyloxy)-4-methoxybenzaldehyde, **54**—dimethyl malonate, **55**—rolipram.

The continuous-flow synthesis of rolipram [58] was demonstrated by the usage of various immobilized heterogeneous catalysts (Scheme 16) in the packed-bed columns. The product was achieved by amine-catalyzed (Si-NH_2_/CaCl_2_; flow rate: 50 µL/min) nitro-aldol condensation with a chiral calcium-catalyzed (CaCl_2_; flow rate: 100 µL/min) asymmetric 1,4-addition reaction, a palladium-catalyzed (Pd/DMPSi/C; flow rate: 100 µL/min) nitro reduction step, and a solid acid-catalyzed (Si-COOH; flow rate: 210 µL/min) hydrolysis/decarboxylation/lactonization sequence. Rolipram was obtained in 50% yield (997.8 mg/24 h). More importantly, both enantiomers of rolipram could be obtained exclusively by changing the column bearing the pyridine bis(oxazoline)-calcium catalyst [59].

### 2.11. Norephedrine

(±)-Norephedrine is a sympathomimetic compound that was first introduced in the 1930s. It has been used widely as a nasal decongestant and an appetite suppressant. It was voluntarily removed from the market in the United States by its manufactures because of concerns raised about its ability to precipitate a stroke when used, or abused, as an appetite suppressant [60].

**Scheme 17:**
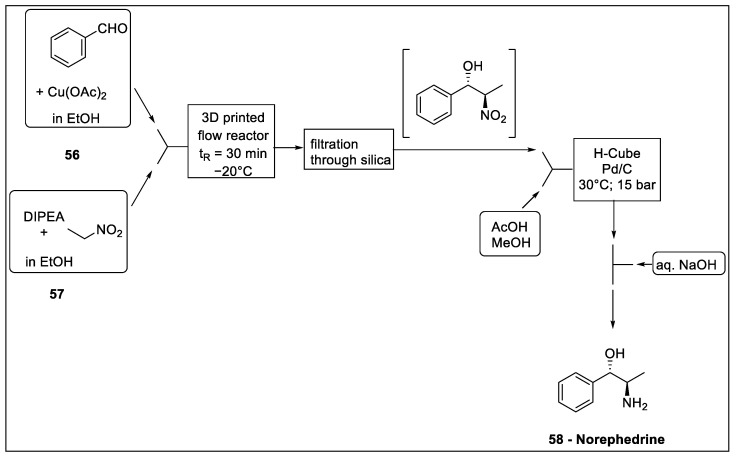
Continuous-flow synthesis of norephedrine: **56**—benzaldehyde, **57**—nitroethane, **58**—norephedrine.

In 2017, Benaglia et al. reported the first cytalytic enantioselective synthesis of norephedrine (Scheme 17) in a homemade 3D-printed flow reactor [61]. The use of 3D-printed reactors, fabricated from different materials (PLA, HIPS, nylon), enabled the rapid screening of devices with different sizes, shapes, and channel dimensions, aimed at the identification of the best reactor set-up. In the extensive study of the synthesis of norephedrine, it was made by injecting the reagents **56** and **57** into the 3D-printed flow reactor at −20 °C for a residence time of 30 min. The output of the reactor was filtered over a pad of silica by elution with EtOH. The resulting mixture was mixed with acetic acid and was subjected to continuous-flow hydrogenation with a H-Cube at 30 °C and a flow rate of 1 mL/min for 2.5 h. After extraction with EtOAc, the product was isolated in 90% yield (productivity not stated) with 78% *ee*, and there was no need for further purification.

For the overview you can find the summary table (Table 1) below for all the evaluated substances and comparison between utilized reactors, their productivities and other parameters.

## 3. Photochemistry

### 3.1. Ibuprofen

**Scheme 18:**
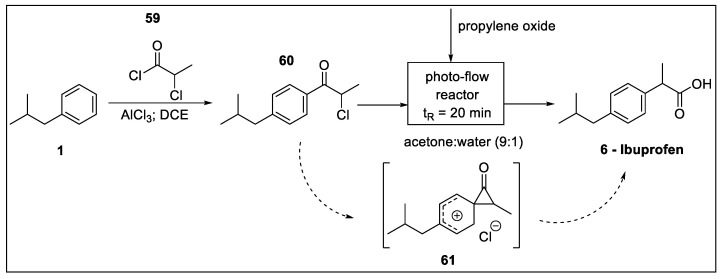
Photochemical synthesis of ibuprofen: **1**—isobutylbenzene, **59**—2-chloropropanoyl chloride, **60**—chloropropiophenone, **61**—transition state, **6**—ibuprofen.

Ibuprofen is one of the APIs which can be prepared either under continuous-flow conditions or photochemically. Based on Baxendale and co-worker’s procedure [62], this compound can be easily prepared under continuous-flow photochemistry conditions. The synthesis (Scheme 18) is based on the photo-Favorskii rearrangement of chloropropiophenone (**60**), using a commercially available coil-flow system for the reaction. Evaluating different reaction conditions, such as residence time (7.5, 10, 15, 20, 30 and 40 min), concentration (0.08, 0.1 and 0.12 vol%), reactor temperature (20, 30, 55, 65, 70 and 75 °C) and 80 W medium-pressure mercury lamps while testing the power setting (80, 90 and 100%) (220–600 nm) with various filters (I–V) reached different emission spectra. It was determined that at the concentration of 0.1%, with 20 min of 80% irradiation at 65 °C, it was possible to generate 76% yield (productivity 2.52 mmol/h) of **6** with a 0.5 mL/min flow rate [14].

### 3.2. Hypericin

Hypericin is a naturally occurring naphtodianthrone found in plants of the genus *Hypericum*, commonly known as Saint John’s Wort [63]. Hypericin has a broad spectrum of pharmacological applications, among which, antidepressive, antiviral, anti-inflammatory and antitumoral activities have been found [64,65,66].

However, obtaining hypericin direct from the plant in both a significant amount and a pure state requires a high commercial cost. In order to overcome this limitation, different synthetic routes have been proposed [67,68,69,70]. The first synthesis by Steglish et al. [71] worked with the procedure consisting of a reductive coupling by a 3-week treatment of emodin (**62**) with alkali and hydroquinone to obtain photohypericin (**63**), which was afterwards irradiated by sunlight to obtain hypericin with 29% yield.

The following works involved a 500 W halogen lamp (588 nm), which after a prolonged irradiation time (overnight) yielded 63% of **61** in the batch set-up [72].

**Scheme 19:**
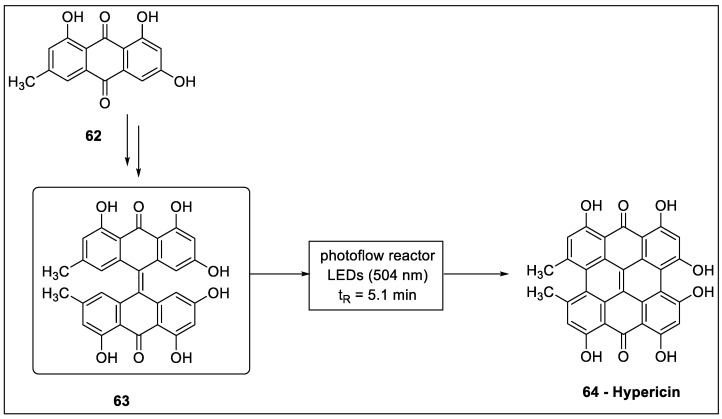
Synthesis of hypericin by utilization of LED light source: **62**—emodin, **63**—photohypericin, **64**—hypericin.

A later work (Scheme 19), consisting of a synthesis of **61** and its derivatives, with the use of high-power (400–1000 W) and multi-frequential light sources in the photochemical step, was described [70,73,74]. The yield of **61** can be significantly improved by using light emitting diodes (LEDs; 504 nm) as a light source. With the flow of 125 mL/s, the product can be obtained in up to 98% yield within 5.1-min reaction time (productivity: 4.85 g/5 min) [75].

### 3.3. Neostenine

The *Stemona* alkaloids, such as neostenine and neotuberostemonine, were reported to have insecticidal, anthelmintic, and antitussive activity against citric-acid-induced cough and various neurochemical effects [76].

**Scheme 20:**
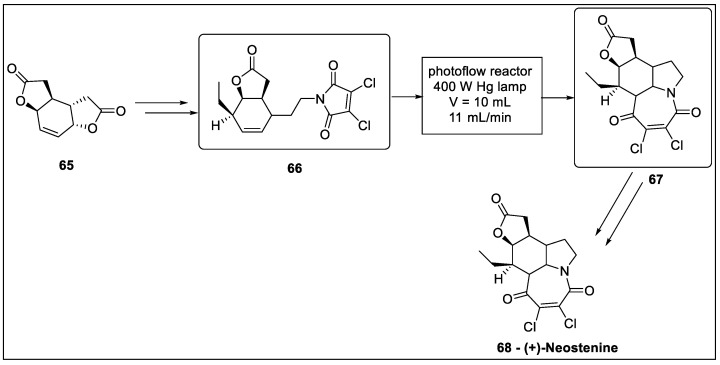
Photocycloaddition synthesis of neostenine: **65**—difuran intermediate, **66**—pyrrole intermediate, **67**—furoindole intermediate, **68**—neostenine.

The key step in the synthesis of neostenine **68** is [5+2] photocycloaddition for the construction of the pyrrolo [1,2-*a*]azepine core. The reaction was initially tested by Booker-Milburn group in a batch [77,78] using a 125 W medium-pressure mercury lamp (300 nm). This particularly sensitive reaction could be performed on a 50 mg scale in a 100 mL immersion-well batch photoreactor giving yields from 40 to 60%. When scaled-up to >100 mg, the yields dropped below 20%. Due to this, the reaction was performed under the flow conditions (Scheme 20) in fluorinated ethylene propylene (FEP) tubing wrapped around a Pyrex immersion well. Irradiation of the solution with a 400 W Hg lamp in a 10 mL volume reactor with the flow rate 11 mL/min allowed the isolation of 63% and the recovery of 20% of the starting material [79]. This enabled the synthesis of 1.3 g of the key photoadduct in a single 9 h run (productivity: 144 mg/h). It would require more than 42 individual batch reactions to reach this amount of material.

### 3.4. Goniofufurone

(+)-Goniofufurone is an example of a styrallactone containing natural products isolated from *Goniothalamus* trees of the plant family *Annonaceae* [80]. Extracts from these plants have been used as traditional medicines in the treatment of edema and rheumatism. Its derivatives have potent antiproliferative effects against a number of human cell lines [81]. 

**Scheme 21:**
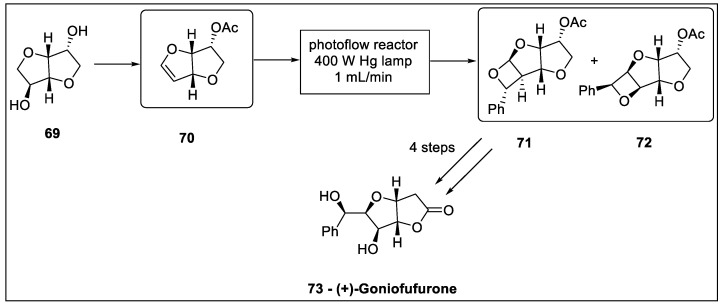
Total synthesis of (+)-goniofufurone: **69**—*D*-isosorbide, **70**—tetrahydrofuro[3,2-*b*]furan-3-yl acetate, **71**—2-phenylhexahydro-2*H*-furo[3,2-*b*]oxeto[3,2-*d*]furan-5-yl acetate, **72**—2-phenylhexahydro-2*H*-furo[3,2-*b*]oxeto[3,2-*d*]furan-5-yl acetate isomer, **73**—goniofufurone.

The Booker-Milburn group [82] developed a short and scalable synthesis of (+)-goniofufurone (**73**) in just five steps from the enantiopure enol ether (**69**) (Scheme 21). The key features include formation of the oxetane ring by a photochemical Paternò–Büchi reaction. Irradiation of tetrahydrofuro [3,2-*b*]furan-3-yl acetate (**70**) in a batch-immersion well with a 400 W medium-pressure mercury lamp (365 nm) gave A 2:1 inseparable mixture of the desired oxetane **72** and structural regioisomer **71**. Despite the product being obtained in a very good yield of 90%; the reaction was slow (24 h run, productivity 0.46 g/h) and required running at a high dilution (0.03 M). This meant that a meaningful scale-up in a batch was rather restricted. The batch limitations of this step were overcome by the use of a three-layer fluorinated ethylene propylene (FEP) flow photoreactor with the utilization of a 400 W medium-pressure lamp. This allowed the synthesis of >40 g of intermediates (93% yield) in a single 83 h run (1 mL/min, 70 min residence time).

### 3.5. Ascaridol

Ascaridol is a bicyclic monoterpene that has an unusual bridging peroxide functional group with anthelmintic [83], sedative and pain-relieving properties and antifungal effects [84]. Ascaridol also showed activity against different tumor cells in vitro [85]. 

**Scheme 22:**
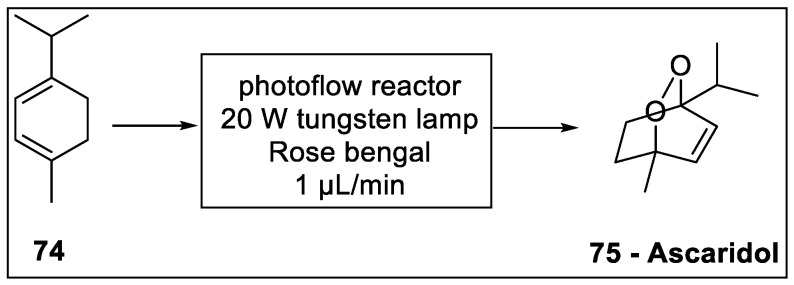
Synthesis of ascaridol: **74**—*L*-terpinene, **75**—ascaridol.

Rose Bengal is employed in the synthesis of ascaridole (**75**) as a photosensitizer [86]. The synthesis can be performed in a microchip reactor equipped with a 20 W tungsten lamp at 550 nm (flow rate: 1 µL/min) for the addition of singlet oxygen to *α*-terpinene (**74**) (Scheme 22). Miniaturizing the reactor’s footprint takes advantage of the small length scales and high surface-to-volume ratios. Additionally, since the channels of the microchip are approximately 50 μm deep, radiation can easily penetrate through the whole reaction environment. Comparison of this microflow reaction to a batch reactor using a 500 W tungsten lamp radiation for 4 h in 100 mL vessel showed that although the microflow reaction provided a higher yield (85% versus 67%), the productivity of the flow reactor was markedly lower (1.5 mg/h versus 175 mg/h). This highlights one common issue with moving to microflow photochemistry: although yields may increase, productivity can be significantly lower due to the capacity of the microchip reactors. This could be overcome by the use of a multiparallel approach [87].

### 3.6. Fulvestrant

Fulvestrant is a chemotherapeutic drug used for breast cancer treatment [88] which contains a perfluorinated side chain synthesized from pentafluoro-1-ol **78** via dehalogenation of pentafluoro-2-iodopentan-1-ol (**77**).

**Scheme 23:**
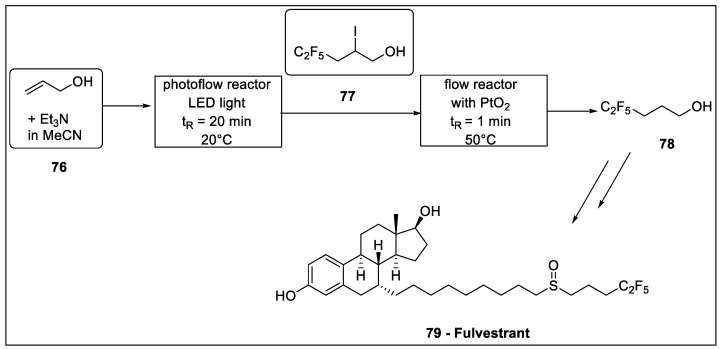
Fulvestrant side chain preparation via continuous-flow photochemistry: **76**—allyl alcohol, **77**—pentafluoro-2-iodopentan-1-ol, **78**—pentafluoro-1-ol, **79**—fulvestrant.

The photochemical step [89] for the intermediate pentafluoro-2-iodopentan-1-ol’s preparation (Scheme 23) was carried out with allyl alcohol (**73**) in acetonitrile with triethylamine (Et_3_N) with the usage of an LED light source (405 nm) and residence time of 20 min (flow: 1 mL/min) at 20 °C. The product was obtained in a good yield of 87%. The next dehalogenation step was carried out in a crude reaction mixture mixed with Et_3_N in a continuous manner using an H-Cube Pro reactor equipped with a PtO_2_ catalyst cartridge. Complete conversion of alcohol **77** into a deiodinated product, **78**, was observed in less than 1 min of residence time (50 °C, 20 bar). Product **78** was obtained in 73% yield in three steps (productivity: 7.6 g/h). Pentafluoro-1-ol is a desired side chain of fulvestrant, **79**.

During commercial development of fulvestrant (**79**), the supply and cost of intermediate pentafluoro-1-ol **78** were noted as the main concerns, which highlight the need for a scalable synthesis, such as this flow methodology [90].

### 3.7. (+)-Epigalcatin

**Scheme 24:**
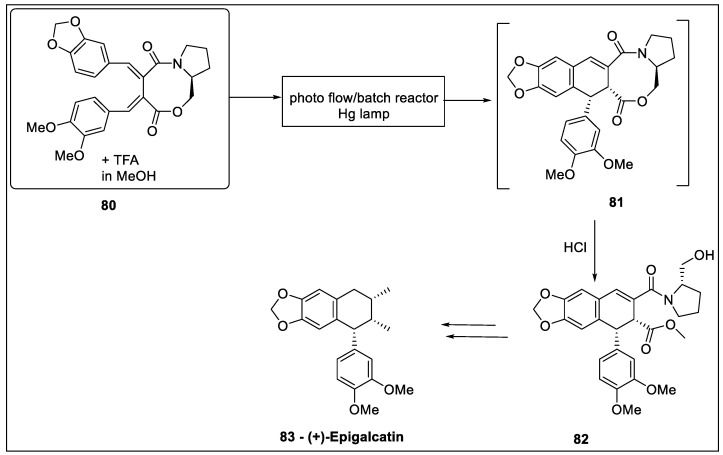
(+)-Epigalcatin photocatalytic preparation: **80**—1,2-bis benzylidene succinate amide ester, **81**—cyclization product—(3,4-dimethoxyphenyl-hexahydro-1*H*-[1,3]dioxolo[4′,5′:6,7]naphtho[2,3-*f*]pyrrolo[2,1-*c*][1,4]oxazocine-6,14-dione), **82**—isolated product—(methyl (5*R*,6*R*)-5-(3,4-dimethoxyphenyl)-7-((*S*)-2-(hydroxymethyl)pyrrolidine-1-carbonyl)-5,6-dihydronaphtho[2,3-*d*][1,3]dioxole-6-carboxylate), **83**—epigalcatin.

Aryltetralin cyclolignans are a family of important products that exhibit various biological properties, such as antiviral, antibacterial and antineoplastic [91]. 

(+)-Epigalcatin can be prepared via a highly stereoselective total synthesis from piperonal in 11 steps (Scheme 24) [92]. The photochemical step was carried out in a microreactor made of a quartz tube, which was multiply folded to form a rectangular reactor (l = 3 m). The synthesis started with α-prolinol as the source of chirality. After four chemical steps, having product **80** in hand, the photochemical step was tested. When performed in a batch, a methanol solution of **80**, with a trifluoroacetic acid (TFA) (0.01 mM) additive, was irradiated for 1 h using medium-pressure mercury lamp (365 nm). The product **82** was obtained in only 21% yield when performed in the batch set-up. Under the continuous-flow photochemical conditions, the product **82** was obtained in 65% yield (productivity: 417 mg/h) by the flow rate of 0.7 mL/min proceeding under the same reaction conditions as the batch procedure. The crucial step of the synthesis—a photocyclization—shows clear advantages in continuous flow over the batch photochemical synthesis.

### 3.8. Myriceric Acid A

Myriceric acid A is a non-peptide endothelin receptor antagonist which was discovered to be a potent vasoconstrictor and is now used in the treatment of pulmonary hypertension [93]. This can be isolated from the bayberry *Myrica cerifera* [94].

**Scheme 25:**
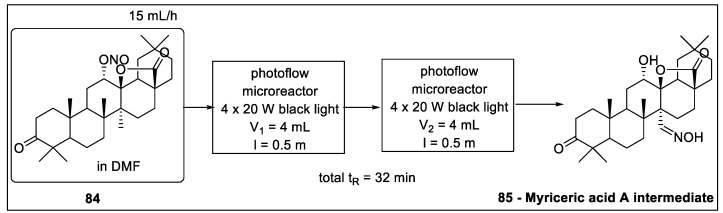
Synthesis of myriceric acid A intermediate: **84**—heptamethyl-3,16-dioxooctadecahydro-(epoxymethano)picen-13-yl nitrite, **85**—myriceric acid A intermediate.

Ryu et al. [95] reported a gram-scale application of nitrite **84** photolysis accelerated in flow in the production of a key intermediate, **85**, en route to myriceric acid A (Scheme 25). The reaction was optimized in a single-channel reactor with either Pyrex or lime soda glass irradiated by a 300 W high pressure mercury lamp (365 nm), which was then swapped for a 15 W black lamp (352 nm). This study demonstrated that doubling the residence time (from 6 to 12 min) allowed the switch of the lamp and decreasing the flow rate from 2 to 1 mL/min. The significant increase in photon efficiency resulted in an increased yield (71% versus 56%). After the optimization, the synthesis was carried out in the serially connected microreactors (1 m total length) with eight 20 W black light lamps. After 20 h of continuous operation, 3.1 g of the desired product was obtained (60% yield; productivity: 155 mg/h).

In a follow-up paper [96], the use of 1.7 W UV-LED (365 nm) was also investigated, and an automated photo-microreactor system employing an array of 48 LEDs yielded 5.3 g (70%; productivity: 132 mg/h) of the product in 40 h under the identical conditions-flow rate of 1 mL/min (residence time 12 min) [79].

### 3.9. Artemisinin

Artemisinin and its derivatives are among the most important drugs against malaria, which is caused by parasitic *Plasmodium* infection. Clinical practice shows that, unlike other antimalarials, artemisinin is active during all the life-cycle stages of the parasite [97]. Whereas these drugs are almost nontoxic to normal cells, several studies have confirmed their potent antitumor activity [98].

**Scheme 26:**
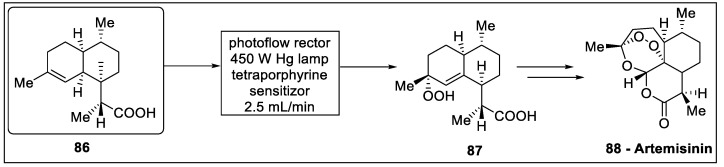
Continuous-flow synthesis of artemisinin: **86**—dihydroartemisinic acid, **87**—hydroperoxy-4,7-dimethyl-octahydronaphthalen-1-yl propanoic acid, **88**—artemisinin.

The total synthesis of artemisinin is too laborious to supply the highly cost-sensitive market. Seeberger and Lévesque [99] reported an innovative and practical synthesis (Scheme 26) under a continuous-flow process from dihydroartemisinic acid, **86** [100]. The continuous-flow system was fabricated in-house with FEP tubing wrapped around a Schenk photochemical reactor containing a 450 W medium-pressure mercury lamp that was cooled to 25 °C. At first, tetraporphyrine (TPP)-sensitized flow photooxidation (flow 2.5 mL/min) of dihydroartemisinic acid (**86**) produced the hydroperoxide **87** in 75% yield and at the rate of 1.5 mmol/min. The photooxidation step was followed by acid-catalyzed Hock cleavage and triplet-oxygen oxidation (continuous-flow under the same conditions), and the last was a series of spontaneous condensations to give artemisinin (**88**) in 45% yield in total [87].

The industrial suitability of this process was demonstrated in 2014, when Sanofi started to exploit this novel semisynthetic route to artemisinin. In the production site of Garessio (Italy), the current production of artemisinin reached 50–60 tons per year, nearly a third of the annual global need [79,101].

### 3.10. Vitamin D3

Vitamin D3, also called cholecalciferol, is a fat-soluble vitamin that helps the body absorb calcium and phosphorus for building and keeping strong bones. Vitamin D is also made by the body when skin is exposed to a sunlight [102].

**Scheme 27:**
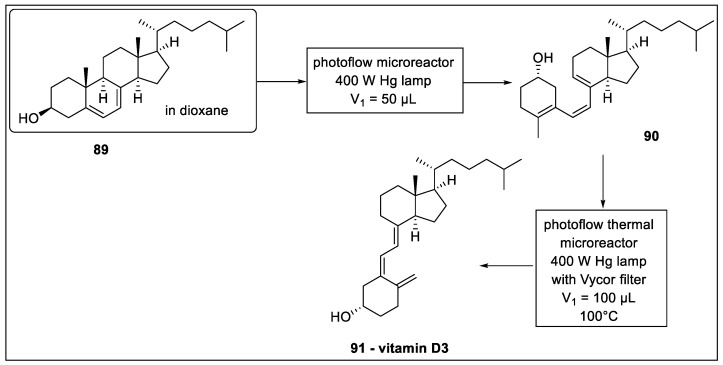
Continuous-flow synthesis of vitamin D3: **89**—provitamin D3, **90**—previtamin D3, **91**—vitamin D3.

The synthesis of vitamin D3 is one of the few industrial photochemical processes that has a yield of less than 20%. Takahashi and co-workers [103] recently induced continuous-flow isomerization of provitamin D3 **89** (Scheme 27), yielding previtamin D3 (**90**) and its isomers in equilibrium [104]. The photochemical conversion to previtamin D3 is induced by a 400 W high-pressure mercury lamp at a wavelength of 360 nm. Consequently, previtamin D3 is photothermally converted into the final product, vitamin D3, at 100 °C in the photoflow thermal microreactor (l = 500 mm) to obtain vitamin D3 (**91**) at 32% overall yield (productivity not stated) after two-stage continuous-flow synthesis (flow rate: 66 µL/min). The authors anticipated that irradiating the reaction mixture during the thermal isomerization by the same light source with the filter to obtain *λ* > 360 nm could improve the yield, since the photoisomerization of the byproducts would shift to produce more previtamin D3 (**89**) [79].

### 3.11. Rosuvastatin

Rosuvastatin is a top-selling drug for the treatment of hypercholesterolemia [105]. 

**Scheme 28:**
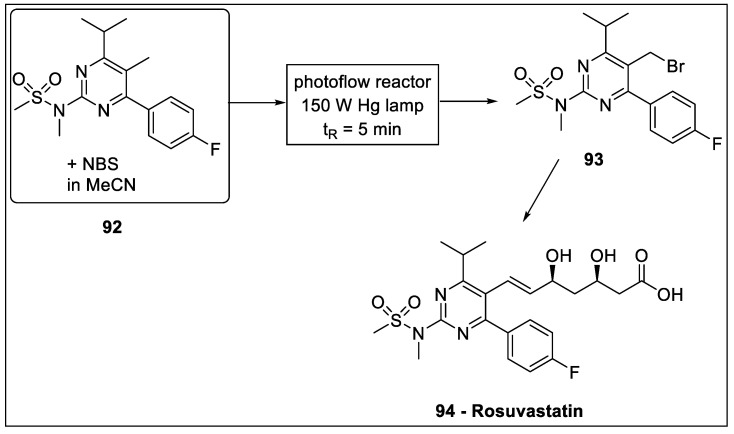
Flow photobromination of an intermediate for the production of rosuvastatin: **92**—5-methyl substituted pyrimidine, **93**—5-methyl brominated pyrimidine, **94**—rosuvastatin.

The batch procedure showed a complete conversion but dealt with the formation of regioisomers and polybrominated byproducts. To better control the rate of bromination and to prevent the formation of the polybrominated byproducts, a photoflow procedure was envisioned [106]. A fluorinated ethylene–propylene (FEP) capillary was coiled around a quartz cooling jacket, and the starting material **92** (Scheme 28) was irradiated with a 150 W medium-pressure mercury lamp (>300 nm) with *N*-bromosuccinimide (NBS). With a residence time of 5 min (flow rate: 3.6 mL/min), the reaction could be completed and provided 58.3 mmol/h of the product (**93**), nearly four times higher than could be obtained in the batch method. No overbromination and a lower overall level of impurities were observed compared to the batch procedure [79]. The intermediate **93** is converted to rosuvastatin (**94**) in the next steps.

### 3.12. Hydantoin

Hydantoin derivatives possess important biochemical and pharmacological properties, for example, as anti-inflammatory agents [107].

**Scheme 29:**
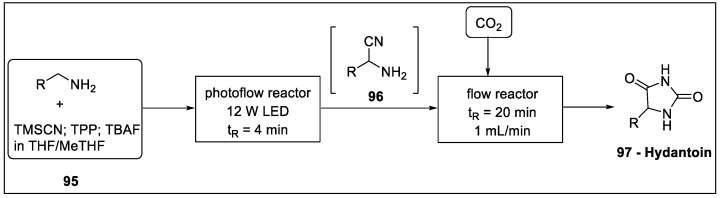
Two-step continuous-flow hydantoin synthesis: **95**—amine, **96**—aminonitrile, **97**—hydantoin.

Taking into account the relevance of this heterocycle, it can be prepared in continuous-flow technology under the principles of green chemistry, carrying out biphasic gas/liquid reactions with an O_2_ and CO_2_ source and with a high-atom economy in connection with light as a traceless reagent and an eco-friendly solvent. Gilmore et al. [108] built hydantoin heterocycle (**97**) with a two-step continuous-flow sequence of photo-oxidation and carboxylation rearrangement (Scheme 29). The first step was carried out photocatalytically in the photoreactor consisting of FEP tubing (inner diameter: 0.76 mm) wrapped around a glass plate in two layers. The 12 W blue LED (420 nm) module was mounted above the photoreactor at a distance of 2 cm. After total residence time of 24 min (flow rate: 1 mL/min), different benzylic and aliphatic unprotected hydantoins were obtained in good yields (52–84%; productivity 3–7.3 g/h) [14].

### 3.13. Oxazolidinone

Oxazolidinones are a very important class of carbonylated heterocycles with widespread application as antibiotics [109,110]. Various oxazolidinones are used as a treatment against infections caused by Gram-positive bacteria that are resistant to other antibiotics. Important examples include linezolid and tedizolid. 

**Scheme 30:**
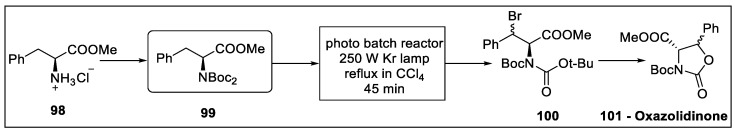
Synthesis of oxazolidinone in batch: **98**—L-phenylalanine methyl ester hydrochloride, **99**—methyl bis(tert-butoxycarbonyl)-*L*-phenylalaninate, **100**—bromo-phenylpropanoate, **101**—oxazolidinone.

Preparation of oxazolidinone as an API precursor has been reported via ring expansion of N-Boc aziridines [111] and cyclization of *β*-chloramines [112]. Both routes used 1,2-amino alcohols as a starting material. In 2006, Crich and Banerjee [113] described a photochemical batch method for the synthesis of oxazolidinone (Scheme 30) from widely available L-phenylalanine methyl ester hydrochloride **98**. After Boc double protection, the double-protected phenylalanine **99** is irradiated with a 250 W Krypton lamp durig refluxing in tetrachlormethan (CCl_4_), providing oxazolidinone (**101**) as a mixture of the 5*R* and 5*S* diastereoisomers in a ratio of 6:1 at 70% yield.

**Scheme 31:**
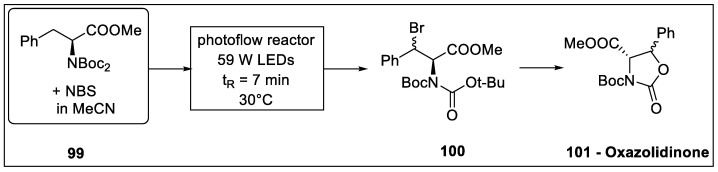
Synthesis of oxazolidinone under the flow conditions: **99**—methyl bis(tert-butoxycarbonyl)-*L*-phenylalaninate, **100**—bromo-phenylpropanoate, **101**—Oxazolidinone.

In 2018, the procedure for oxazolidinone synthesis was optimized by the Kappe group [114] under flow-photochemical conditions (Scheme 31). The following reaction conditions were optimized: residence time, temperature, N-Bromosuccinimide (NBS) equivalents, concentration, wavelength and power of irradiation. This led to a scalable continuous procedure, which completes the photochemical step within 10 min of residence time and 75% yield (flow: 0.2 mL/min), utilizing a commercially available photochemical flow reactor (Vapourtec UV-150) with 60 W LED (*λ* 365 nm). This procedure was further scaled-up again in the commercial system (Corning^®^ Lab Flow photoreactor), which consisted of a glass-chip (2.7 mL volume) surrounded by two LED panels positioned either side of the chip. The most favorable results were achieved when 395 nm LEDs were utilized with a flow rate of 0.8 mL/min (residence time: 3.46 min), providing an total isolated yield of 94% and a productivity of 90 mmol/h.

### 3.14. CDK9 Inhibitor

Cycline-dependent kinase 9 (CDK9) is critical for RNA polymerase transcription initiation, elongation and termination in several key biological processes, including development, differentiation and cell-fate responses [115]. The compound discussed in the following paragraph inhibits cycline-dependent kinase 9 at a nanomolar concentration, making it a potent API for a potential cancer drug [116].

**Scheme 32:**
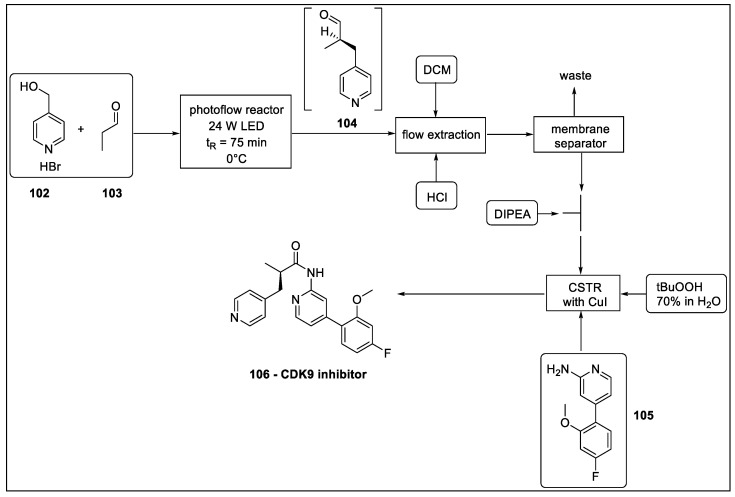
Continuous-flow synthesis of CDK9 inhibitor: **102**—pyridin-4-ylmethanol, **103**—propionaldehyde, **104**—(*R*)-2-methyl-3-(pyridin-4-yl)propanal, **105**—4-(4-fluoro-2-methoxyphenyl)pyridin-2-amine, **106**—CDK9 inhibitor.

In 2022, Benaglie et al. reported the synthesis of CDK9 inhibitor (Scheme 32) under highly enantioselective in-flow protocol [117]. After figuring out the best performing LEDs, a homemade, custom-designed photoreactor was benchmarked under batch conditions to be later compared to the continuous-flow processes. After running some successful tests under batch conditions, this methodology was incorporated into the continuous-flow process, and finally the fully continuous, fully telescoped process to synthesize a complex API was realized. The final process consisted of four stages: asymmetric photobenzylation under continuous flow, inline continuous work-up, neutralization and the final oxidative imidation. The photoflow reaction between pyridin-4ylmethanol (**102**) and propionaldehyde (**103**) was performed in PFA tubing (l = 1.9 m; internal diameter: 0.02 inch; flow rate: 5.13 μL/min) wrapped around an LED light source (395 nm), which was sealed inside a Pyrex glass tube. This was followed by extraction with DCM in the presence of diluted HCl. The next step was flow neutralization by diisopropylethylamine to pH 7. The output of this step was connected to the continuous stirred-tank reactor (CSTR), containing a filtering paper bag loaded with CuI. The CSTR led to the inlet of the amine **105**. The CSTR output was worked up with EtOAc in a mixture with Na_2_SO_3_. The final product was obtained in 23% overall yield and 95% *ee* after preparative TLC.

For the overview you can find the summary table (Table 2) below for all the evaluated substances and comparison between utilized reactors, their productivities and other parameters.

## 4. Comparison of Flow and Photochemical Approach to Ibuprofen Synthesis

In conclusion, continuous-flow synthesis and flow-photochemical methodology are compared for the high-volume drug ibuprofen. For comparison reasons, the newest flow approach (Jamison, 2015; see Scheme 2) was chosen, along with the flow-photochemistry process (Baxendale, 2013; see Scheme 18).

In this comparison (Table 3), the flow approach to a synthesis of ibuprofen provides better results regarding hourly output (8.09 vs. 0.52 g/h), overall yield (83 vs. 76%) of the material, total residence time for the synthesis (3 vs. 20 min) and cost (2822 vs. 5917 USD/kg). The prices for these calculations include only the costs of reagents and solvents, based on the equivalents and volumes needed for each procedure. The operating and acquisition costs were not taken into account. The twice-higher price of the photochemical approach is mostly caused by the expensive chloropropionyl chloride (1030 USD/kg). All the prices for the calculation were taken from the commercial supplier Sigma Aldrich. This does not mean that the flow approach must be better in all the cases, but for ibuprofen synthesis it is. What can be said is that these two procedures provide better results than a batch method in most cases, as depicted in the previous chapters—for example, for linezolid, rufinamid, hypericin or epigalcatin synthesis—considering the reaction time, yields, productivity or safety aspects.

## 5. Conclusions

Many advantages of synthesis in flow and photochemistry have been demonstrated. Flow synthesis helps to overcome the limitations that would have been imposed by conducting an analogous synthesis in batch synthesis. These limitations can be, for example, minimizing the safety hazards by avoiding both accumulation and isolation of hazardous intermediates. The flow technology can overcome the purity issues and others. While this methodology is very useful and promising, the implementation is not straightforward, and it needs to be tailored specifically case by case.

A flow methodology can be easily combined with photochemistry, which means utilization of irradiation of the reaction mixture, either with or without the presence of the photocatalyst. The irradiation can be performed by low-, medium- or high-pressure lamps; and nowadays, more often by LED sources, which show high monochromaticity and high intensity of the light source.

Both of these methods show advantages over batch procedures considering purity, yield and reaction duration.

Photochemistry has recently seen a remarkable increase in researchers’ attention. The first reason is the use of continuous-flow reactors, which give a great degree of operational flexibility in handling such photochemical reactions. The second reason is that reactions could be carried out in a highly selective and mild fashion (room temperature, visible light and avoidance of toxic chemicals). In this context, the combination of flow and photochemistry is an excellent approach that has been successfully employed in recent years. The views expressed herein are the writers’ views only and not those of any company, employer or organization associated with the writers.

## Figures and Tables

**Table 1:** Summary table of flow preparations of APIs.

Substance	Set-Up	Flow Rate	Residence Time [min]	Productivity [mg/h]	Reactor	Source
Ibuprofen	Flow	151–260 µL/min	10	540	3× PFA microflow reactor in fully continuous process with off line purification	[21]
	Flow	109–500 µL/min	3	8 g/h	3× PFA microflow reactor	[16]
Warfarin	Flow	1 µL/min	10	NA	Flow PEEK microreactor	[11]
	Flow	4 µL/min	10	NA	4 PEEEK microreactors in parallel	[22]
Atropine	Flow	8–350 μL/min	15.2	48	2 flow reactors with three sequential in-line separations	[27]
	Flow	87.5 µL/min209 µL/min	3.5 24	996	2 flow reactors with in-line separation	[28]
Ketamine	Batch	-	-	-	Batch reactor	[31]
	Flow	0.25 mL/min0.43 mL/min	5 2	NA	2 flow reactors and 1 packed K10 bed	[33]
Imatinib	Flow	0.1–0.4 mL/min	30	64	Multistep flow process with packed beds, in-line purification and inline solvent switch	[36]
	Flow	5–143 µL/min	48	327	Three-step one-flow synthesis without solvent switch	[37]
Rufinamide	Batch	-	-	-	Batch reactor	[40,41]
	Flow	2–41 µL/min	6	217	2 PFA microreactors and one copper microreactor	[44]
	Flow	1.2 or 1.8 mL/min	5.5 or 20	237 (convergent)22 (linear)	Automated synthesis, novel radial system	[46]
Oseltamivir	Batch	-	-	-	Batch reactor	[48]
	Flow	0.1–0.4 mL/min	310	4	4 flow reactors and Zinc/Celite packed bed with low stability	[49]
Linezolid	Batch	-	-	-	Batch reactor	[51]
	Flow	55–186 µL/min	27	816	6 flow reactors and Pd packed bed	[52]
Lomustine	Flow	12–50 µL/min	9	110	Two flow steps and in-line extraction	[55]
Rolipram	Flow	50–210 µL/min	NA	42	Flow process with various immobilized heterogeneous catalysts	[58]
Norephedrine	Flow	1 mL/min	180	NA	3D-printed flow reactor	[61]

NA—not available in literature.

**Table 2:** Summary table of photochemical preparations of APIs.

Substance	Set-Up	Light Source	Wavelenght [nm]	Power [W]	Productivity	Reactor	Source
Ibuprofen	Flow (0.5 mL/min)	Medium pressure Hg lamp	220–600	80	520 mg/h	Commercially available system with various light filters	[62]
Hypericin	Batch	Sun	Full spectrum	-	-	Batch reactor	[71]
	Batch (overnight)	Halogen lamp	588	500	-	Batch reactor	[72]
	Flow (125 mL/min)	LED	504	NA	4.85 g/h	Photoflow reactor	[75]
Neostenine	Batch	Medium pressure Hg lamp	>300	125	-	Batch reactor	[77,78]
	Flow (8 mL/min)	Hg lamp	280–300	400	144 mg/h	FEP tubing wrapped around pyrex immersion well	[79]
Goniofufurone	Batch (24 h)	Medium-pressureHg lamp	365	400	-	Batch reactor	[82]
	Flow (1 mL/min)	Medium-pressure Hg lamp	365	400	460 mg/h	FEP flow tubing	[82]
Ascaridol	Batch (4 h)	Tungsten lamp	550	500	-	Batch reactor	[86]
	Flow (1 µL/min)	Tungsten lamp	550	20	1.5 mg/ 175 mg/h	Microchip flow reactor,	[86]
Fulvestrant	Flow (1 mL/min)	LED	405	NA	7.6 g/h	1 photo and 1 flow reactor with PtO_2_	[89]
Epigalcatin	Batch (1 h)	Mediumpressure Hg lamp	365	NA	-	Batch reactor	[92]
	Flow (0.7 mL/min)	Medium pressure Hg lamp	365	NA	417 mg/h	Flow photoreactor	[92]
Myriceric acid A	Flow (2 mL/min)	High-pressure Hg lamp	365	300	NA	Single-channel microreactor	[95]
	Flow (1 mL/min)	Black light	352	15	155 mg/h	2 flow microreactors in series	[95]
	Flow (1 mL/min)	LED	365	1.7	132 mg/h	Automated photo microreactor system	[96]
Artemisinin	Flow (2.5 mL/min)	Medium-pressure Hg lamp	>300	450	424 mg/h	Photoflow reactor with TPP photosensitizer	[99]
Vitamin D3	Flow (66 μL/min)	High-pressure Hg lamp	360	400	NA	1 photo and 1 flow thermal microreactor	[103]
Rosuvastatin	Flow (3.6 mL/min)	Medium pressure Hg lamp	>300	150	28 g/h	FEP capilar coiled around the quartz well	[106]
Hydantoin	Flow (1 mL/min)	LED	420	12	3–7.3 g/h	Two-step continuous-flow sequence	[108]
Oxazolidone	Batch (45 min)	Krypton lamp	NA	250	-	Batch reactor	[113]
	Flow (0.2 mL/min)	LED	365	60	NA	Commercially available photoflow reactor	[114]
	Flow (0.8 mL/min)	LED	395	60	29 g/h	Commercial system with glass chip surrounded by two LED panels	[114]
CDK9 inhibitor	Flow (5.13 μL/min)	LED	395	12	NA	PFA tubing wrapped around a LED light source sealed inside a Pyrex glass tube	[117]

NA—not available in the literature.

**Table 3:** Comparison of flow and photochemical approaches to ibuprofen synthesis.

Approach	Advantages	Disadvantages	Yield [%]	Total Cost (USD/kg) *
Flow approach	-Fast (total *t_R_* = 3 minutes) -High-throughput (8.09 g/h)	-Reaction system prone to clogging	83	2822
	-Handles dangerous and corrosive reagents	-Double pumping system required		
	-Available and inexpensive reagents			
Photochemistry approach (micro-flow)	-Photo-reactor compatible with existing flow-system	-Low productivity(520 mg/h)	76	5917
	-Allows a real time analysis when linked to photo-spectrometer	-Heat generated by the lamp		
	-Commercially available system with various light filters			

* prices for the calculation adopted from commercial supplier Sigma Aldrich.

## Data Availability

Not applicable.
